# Randomised controlled trial of a baked egg intervention in young children allergic to raw egg but not baked egg

**DOI:** 10.1186/s40413-017-0152-5

**Published:** 2017-06-16

**Authors:** Merryn Netting, Michael Gold, Patrick Quinn, Adaweyah El-Merhibi, Irmeli Penttila, Maria Makrides

**Affiliations:** 1 Children’s Nutrition Research Centre, South Australian Health Medical Research Institute, 72 King William Road, North Adelaide, South Australia 5006 Australia; 2 Discipline of Paediatrics, The University of Adelaide, Adelaide, Australia; 3 School of Medicine, The University of Adelaide, Adelaide, Australia; 4 Department of Allergy and Immunology, Women’s and Children’s Health Network, Adelaide, Australia

**Keywords:** Egg, Egg allergy, Baked egg, Food allergy, Oral tolerance, Randomised controlled trial

## Abstract

**Background:**

Consumption of baked egg by raw egg allergic children is associated with immune changes suggesting development of tolerance. However, causation has not been tested using a double blind randomized controlled trial (RCT). We aimed to compare clinical and immunological outcomes after baked egg (BE) consumption in young BE tolerant egg allergic children.

**Methods:**

In a double blind RCT, BE tolerant egg allergic children consumed 10 g BE (1.3 g protein) 2 to 3 times per week for 6 months (*n* = 21 intervention group) or similar egg free baked goods (*n* = 22 control group) while maintaining an otherwise egg free diet. The final assessment was a raw egg oral food challenge (OFC) 1 month after ceasing the intervention product. Egg specific IgE and IgG4 were assessed at baseline and 7 months.

**Results:**

After the intervention there was no difference in raw egg tolerance between groups, (23.5% (4/17) intervention group and 33.3% (6/18) control group). This was independent of age and amount of BE consumed (aOR 0.50 CI 0.11–2.40 *p* = 0.39). Both groups demonstrated decreased egg specific serum IgE titres and decreased whole egg specific IgE/IgG4 ratios.

**Discussion:**

We conducted this trial because inclusion of baked egg protein in the diet of egg allergic children appears to move children towards a more tolerant immune profile. Strengths of our study include design of the blinded intervention, the consistent dosing protocol and the regular monitoring of symptoms and intake. However, the study was limited by small sample size resulting in insufficient power to show statistically significant results.

**Conclusion:**

Our study suggests that short term, regular consumption of BE by BE tolerant 1 to 5 year old children with IgE mediated raw egg allergy may not induce, accelerate or slow development of tolerance to raw egg in this selected population. Trials with larger sample sizes are required to further test this hypothesis.

**Trial Registration:**

The trial was registered on 7^th^ February 2012 with the Australian New Zealand Clinical Trials Registry (ACTRN 12612000173897).

## Background

Many egg allergic children tolerate baked egg (BE) before less well-cooked forms of egg as heating causes structural changes in some egg epitopes and there is a matrix effect when egg is baked with wheat that may also be important [1–4]. Inclusion of BE in the diet of egg allergic children, when tolerated, has become accepted clinical practice [5–8] and it is reported that individuals consuming BE tolerate lightly cooked egg earlier than those not consuming BE [9, 10]. Regular consumption of BE is associated with increases in specific IgG4, and decreases in specific IgE [1, 11], immunological changes similar to those observed during specific oral tolerance induction (SOTI) [12]. From this it was hypothesised consumption of BE could promote tolerance to uncooked egg protein [13].

It remains unclear, in children with raw egg allergy tolerant to BE if ingestion of BE accelerates tolerance acquisition to raw egg as randomised controlled clinical trials (RCT) have not been undertaken. It is also not clear from observational studies whether BE tolerant children who gain raw egg tolerance earlier than those not tolerant to BE were moving towards natural resolution of their egg allergy, or if this was due to changes with time or other unidentified confounders [1, 11].

Our study’s primary aim was to determine whether raw egg allergy is better resolved by regular consumption of BE (intervention group) compared with an egg free diet (control group). We also examined the effect of regular BE exposure on immunity, particularly patterns of evolving allergen-specific responses.

## Methods

### Study design

Six month old to five year old children with IgE mediated egg allergy following egg free diets were recruited from the Women’s and Children’s Hospital Allergy Clinic. Following recruitment all children had egg allergen SPT (see Appendix 1). Egg allergy was defined as children with a convincing clinical reaction to egg within the past 12 months and evidence of current sensitisation on the basis of positive SPT to egg white *or* evidence of current sensitisation consistent with a >95% likelihood of clinical reactivity (SPT to egg white ≥ 5 mm if aged under 2yo, or ≥ 8 mm in children aged 2 to 5yo) [14, 15]. To assess BE tolerance, all children had an open, medically supervised BE oral food challenge (OFC) (10 g of egg in a muffin) [16]. BE tolerant children with EW SPT <5 mm (6 months to 2yo) or <8 mm (2 to 5yo), who reported no clinical reactions to raw egg in the previous 12 months had an open pasteurised raw egg OFC [17] to confirm they still had a raw egg allergy.

The study was conducted using a double-blind, randomised, placebo controlled trial design. Baseline characteristics including demographics, allergy history and anthropometrics (weight and length or height) were gathered. For those children with eczema the clinical severity was scored using the SCORAD assessment [18]. A peripheral blood sample was collected to measure baseline whole egg (WE), egg white (EW), ovalbumin (OVA) and ovomucoid (OVM) specific IgE (sIgE), WE specific IgG4 (sIgG4) and functional cell response profiles. Written informed consent was obtained before trial participation. Approval was granted by the local institutional review board (Human Research Ethics Committee; REC2400/9/14) of the Women’s and Children’s Health Network Adelaide, Australia, and the trial registered with the Australian New Zealand Clinical Trials Registry (ACTRN 12612000173897).

### Randomisation and concealment allocation

Each child was randomly assigned to the intervention group or the control group using a computerised randomisation schedule stratified by age (6 months to 2.5 years and 2.6 years to 5 years). A research assistant (with no involvement in the outcome assessments) was responsible for baking and coding the dietary products for the trial.

### Dietary intervention

The study compared the effects of inclusion of baked egg containing (intervention group) or egg free (control group) products in the diet of raw egg allergic children for 6 months after randomisation. Both groups maintained an otherwise egg free diet with study muffins, biscuits (cookies) or cake offered to the child for consumption 2 to 3 times per week for 6 months. The intervention group consumed 10 g BE (1.3 g egg protein) per serve. The control group consumed egg free products tested for similarity in terms of appearance, taste, and texture. Intervention products were offered 2 to 3 times a week (consistent with the Australian Dietary Guidelines for inclusion of ‘discretionary foods’ in a child’s diet [19]).

To assess compliance with the intervention, care givers maintained an intake and symptom diary, participants were reviewed 1 month after randomisation and telephoned monthly for the study duration.

After 6 months the intervention was stopped and children continued an egg free diet for an additional month, to differentiate between desensitisation and development of sustained unresponsiveness to egg [20].

Adverse events were defined as flares in eczema, urticaria, angioedema or vomiting associated with intake of the intervention product or hospitalisation for any reason greater than 24 h. Serious adverse events (defined as any death, admission to intensive care, or anaphylactic reaction) were reported to the institutional review board.

### Outcome assessments

The primary outcome was raw egg allergy 1 month after the intervention ended, assessed by an open medically supervised, graded pasteurised raw whole egg OFC [17, 21]. A positive reaction to an OFC was defined by symptoms within 2 hours of the OFC according to the PRACTALL Guidelines [22, 23]. SPT to EW was performed prior to the OFC.

### Analysis of immune outcomes

To assess sensitisation egg allergen (WE, EW, OVA and OVM) sIgE levels were measured. WE sIgG_4_ was used as a marker of tolerance. The sIgE and sIgG4 were analysed at the end of the trial (maintaining blinding of the sample ID). T cell surface markers and Th1/Th2 cytokines were also analysed (see Appendix 2).

### Statistical analysis

A sample size estimate was calculated based on the known natural history of egg allergy expecting after 6 months of management with an egg free diet 90% of children would still be egg allergic [2]. We hypothesized regular exposure to BE would result in 30% absolute reduction (ie from 90 to 60%) of egg allergy. To detect such a difference with 90% power and *p* = 0.05, we estimated we would need 49 children per group (total *n* = 98) and aimed to recruit 55 children to each group to allow for withdrawals from the study. However, after screening 83 children for BE tolerance over 20 months, and enrolling 43 participants, screening for the study ceased due to resource constraints.

Analyses were performed according to the randomized group using STATA 13.1 (StataCorp LP) or the InStat program v 6.05 (Graph Pad software, USA). Statistical significance was assessed at the 0.05 level. The proportion of children tolerant to egg at the study’s end was compared between groups. Secondary comparisons between groups included changes in sIgE and sIgG4 results and other immune outcomes. For sIgE and sIgG4 results, standard linear regression was performed including the baseline level as a covariate to ensure estimated differences between groups were not biased due to differences in baseline wheal size and/or regression to the mean effects. For ‘adjusted’ analyses, age stratum was also included. In all cases, sensitivity analyses (removal of outlying/influential observations) were undertaken, and did not affect the conclusions. Change between groups was assessed using the Wilcoxon Rank-Sum Test.

For other immune outcomes a non-parametric approach was used for analysis because of highly skewed distributions of all variables and small sample sizes.

## Results

Randomization occurred from 22 May 2012 to 20 January 2014. The final follow up appointment was completed on 8 October 2014. While we intended to screen from 6 months of age many babies could not tolerate the texture of baked egg, so screening challenges were scheduled from 1 year of age. The outcomes of the screening BE OFCs are in Appendix 3.

**Fig. 1:**
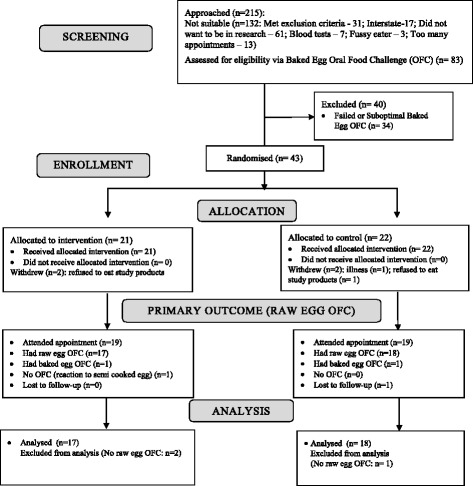
Study participant flow diagram. OFC = Oral food challenge. A Suboptimal baked egg oral food challenge was defined as failure to consume an adequate amount of the challenge food

We randomised 43 children (*n* = 21 intervention group; *n* = 22 control group), aged 1.0 to 5.3 years. Both groups had similar family and clinical backgrounds (Table 1). Four parents withdrew consent and 38 children (19 from each group) attended primary outcome assessments (Fig. 1).

**Table 1:** Demographic and clinical characteristics of children at study entry

Characteristic	Baked egg group *n* = 21	Control group *n* = 22
Maternal age (years)^a^	35.67 (3.7)	34.14 (3.7)
Maternal ethnicity Caucasian^b^	16 (76%)	19 (86%)
Age at screening (years)^c^	2.00 (1.21–3.25)	2.13 (1.29–3.12)
Male sex^b^	14 (67%)	16 (73%)
First degree relative with atopy^b^	18 (86%)	18 (82%)
Birth weight (grams)^a^	3509 (538)	3592 (470)
Gestational age at birth (weeks)^a^	38.9 (1.0)	38.7 (1.0)
Ever breastfed?^b^	21 (100%)	20 (91%)
Breastfed at screening?^b^	2 (10%)	2 (9%)
Age at diagnosis of egg allergy (months)^a^	9.5 (4.3)	7.7 (3.4)
History of anaphylaxis to egg?^b^	3 (14%)	5 (23%)
Other IgE mediated food allergies^b^	15 (71%)	18 (82%)
Eczema^b^	15 (71%)	19 (86%)
Eczema severity (Objective SCORAD score)^c^	1.80 (0.00–12.33)	3.90 (0.00–9.0)
Asthma (Doctor diagnosed)^b^	2 (9.5%)	6 (27%)

Values are presented as follows: ^a^mean (SD), ^b^number (percentages) or ^c^median (IQRs)

## Clinical outcomes

### Tolerance to raw egg after the intervention

Thirty-five children had raw egg OFCs (*n* = 17 intervention group; *n* = 18 control group), (Fig. 1). 23% (4/17) children from the intervention group and 33% (6/18) control group passed the raw egg OFC and were therefore egg tolerant. There was no difference between groups in likelihood of tolerating raw egg (Odds Ratio [OR], 0.62; 95% CI, 0.14–2.73; *p* = 0.52), even after age adjustment (OR 0.50; CI, 0.11–2.40; *p* = 0.39).

Per protocol analysis comparing children consuming 2 to 3 serves per week of study product 42% (4/19, intervention group), 68% (13/19, control group) demonstrated no difference between groups in the proportion passing the raw egg OFC (OR, 1.2; CI, 0.185–7.77; *p* = 0.85). Adjusted analysis was not performed because of small numbers.

There were three protocol deviations due to clinical decisions not to proceed with the end of study raw egg OFC. Two children who had refused to eat the study product had high EW SPT values (intervention group *n* = 1, 12.5 mm wheal; control group *n* = 1, 24.5 mm wheal) and instead of raw egg OFCs had BE OFCs (passed). It is not known if these children would have passed the raw egg OFC if given. One child (intervention group) did not have a raw egg OFC due to an accidental reaction to semi-cooked egg the week prior to the appointment, and would have reacted to the raw egg OFC. Inclusion of this child in the analysis would not change the final outcome of the trial.

### Compliance with the intervention

Participants were offered 2324 serves of intervention product during the study. Intervention group children were offered fewer (1065) serves, consuming a median of 1.6 (IQR 0.7–2.6) serves per week, compared with the control group offered 1259 serves, consuming 2.3 (IQR 1.4–2.7) serves per week. The differences between groups were not significant.

### Compliance with the egg free diet

One child (intervention group) had several accidental exposures to egg during the course of the study. This child did not have the end of study raw egg OFC due to a recent reaction to semi-cooked egg. No other accidental exposures to egg were reported by participants in the study.

### Adverse events

Nine children in the intervention group and eight children in the control group reported adverse events (Table 2). One child (control, egg free group) was diagnosed with Eosinophilic Oesophagitis during the study. Three serious adverse events were reported (anaphylaxis treated with adrenaline during the raw egg OFC [*n* = 2 intervention group; *n* = 1 control group]).

**Table 2:** Adverse events

	Intervention group			Control group		
	Related to ingestion of the study product			Related to ingestion of the study product		
	No	Yes	Unclear	No	Yes	Unclear
Urticaria	2	1				
Eczema flares	2		1	5		
Vomiting	1	2^a^		3		
Abdominal Pain	1					
Hospital Admission >24 h	1			2		

^a^vomiting in both cases was associated with reintroduction of study product after gastroenteritis

## Immunological outcomes

### Egg allergen specific IgE results

**Fig. 2:**
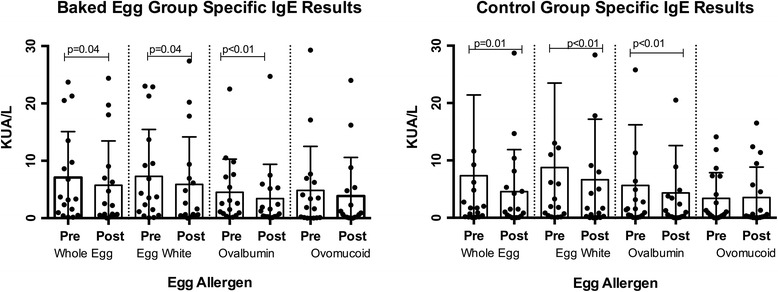
Changes in egg specific IgE from baseline to end of intervention. Changes in egg allergen specific IgE levels from the beginning to end of the intervention. Box and bars indicate the median and IQR

Egg allergen sIgE levels did not differ between intervention groups from baseline to end of the intervention when compared using linear regression analysis for effect of group allocation on outcome, adjusted for age stratum. For both groups we observed significant reductions with time in median specific IgE levels (kUA/L) to WE, (BE group 3.33 (0.75–11.62) to 2.20 (0.47–8.01), *p* = 0.04; control group 1.71 (0.19–6.94) to 1.26 (0.19–6.01), *p* = 0.01), EW (BE group 3.72 (0.82–11.62) to 1.61 (0.42–8.17), *p* = 0.04; control group 1.95 (0.28–11.60) to 1.66 (0.14–8.55), *p* < 0.01) and OVA sIgE (BE group 2.57 (0.64–7.40) to 1.23 (0.29–5.23), *p* = 0.01; control group 1.48 (0.33–5.29) to 0.75 (0.15–4.05), *p* < 0.01) (Fig. 2).

### Whole egg serum specific IgG4 and IgE to IgG4 ratio results

**Fig. 3:**
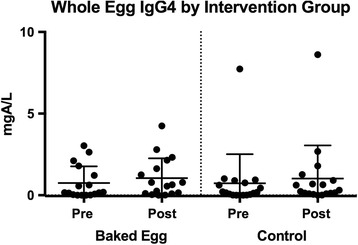
Changes in whole egg specific/IgG4 from baseline to end of intervention. Changes in whole egg specific IgG4 from the beginning to end of the intervention. Median and IQR indicated

There was no significant difference in WE sIgG4 levels within the intervention and control groups or between the groups from baseline to end of the intervention. [BE group (0.16 (0.02-1.5) to 0.64 (0.10-1.88), *p* = 0.3); control group (0.16 (0.01–0.79) to 0.27 (0.09–0.99), *p* = 0.3)] (Fig. 3).

**Fig. 4:**
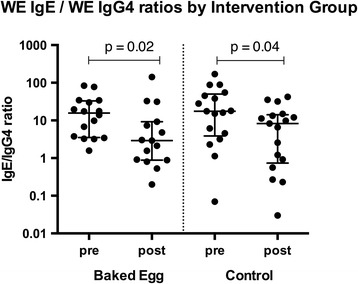
Changes in whole egg specific IgE/IgG4 ratios from baseline to end of intervention. Changes in whole egg specific IgE / IgG4 ratio from the beginning to end of the intervention. Median and IQR indicated

No difference in median WE specific IgE/IgG4 ratio was observed between groups during the intervention. However, a significant decrease in the median WE IgE/IgG4 ratio (indicative of evolving tolerance to egg) was observed with time in both BE intervention (15.63 (3.51–35.00) to 2.91 (0.88–9.21), *p* = 0.02) and control groups (17.50 (3.86–50.18) to 8.28 (0.74–14.13), *p* = 0.04) (Fig. 4).

### Cellular immune analysis

We assessed Th1/Th2 cytokines and T cell phenotypes, with no significant difference observed between groups (data not shown).

## Discussion

This double blind, placebo controlled RCT compares the effect of consumption of BE (with avoidance of all other forms of egg) in 1 to 5 year old BE tolerant, raw egg allergic children on the development of tolerance to raw egg. In our study group, development of tolerance to raw egg was independent of consumption of BE. We conducted this trial because inclusion of baked egg protein in the diet of egg allergic children appears to move children towards a more tolerant immune profile [13]. In addition inclusion of baked proteins in the diet, when tolerated, improves quality of life.

Strengths of our study include design of the blinded intervention, the consistent dosing protocol and the regular monitoring of symptoms and intake. Randomized groups were of similar age, allergy background and egg allergy phenotype, and the timing of assessments for clinical and immunological outcomes add additional strength to our results. We acknowledge that the small sample size is a major limitation because of the resulting insufficient power to show statistically significant results. Over 200 families of egg allergic children were approached regarding the trial, and of the children screened for the trial, only half tolerated BE, which was less than expected [1, 24]. There are several potential explanations for the lack of difference between the two trial arms:

- Small sample size, and thus the lack of difference may be due to chance. Whilst our study was underpowered, we did not see trends suggesting any effects on either clinical or immunological outcomes.
- The time period of the intervention may have been too short. However, decreased SPT wheal sizes and egg sIgE, and increased sIgG4 have been reported after 3 to 6 months exposure to BE [1, 11], leading to conjecture that inclusion of BE, when tolerated, in the diets of egg allergic children may modulate the immune system [8, 13, 25, 26]. In our group of BE tolerant children, there was no difference between groups in reduction in egg allergen sIgE levels or increase in WE sIgG4 levels. Our results are consistent with Tey et al. [27], who reported no difference in the rate of decline in EW SPT wheal size in 3 to 6 year old egg allergic children consuming BE compared with an egg free diet indicating that this change may be independent of consumption of BE.
- The dose of BE may have been too low, or too infrequent. To comply with Australian healthy eating guidelines related to consumption of ‘discretionary foods’ [19] we asked the children to consume the study foods 2 or 3 times per week. This dose rate is consistent with the maintenance phase of several egg SOTI studies [28–30], but less frequent than Lemon-Mule et al. [1] who dosed 1 to 3 times daily. In our study, three children (6%) (*n* = 2 intervention group; *n* = 1 control group), refused to consume intervention products possibly due to finicky eating, or related to the texture of the study products. Poor compliance has also been reported in other immunotherapy trials [31, 32], including other BE trials [33], and also was recently reported in the EAT study[34]. It is possible that some children in our study may have lost tolerance to BE, reflected by refusal to consume the study product. Development of symptoms when consuming BE and subsequent refusal to consume BE has been reported in children passing BE OFCs [16]. Participants in our study kept an intake and symptom diary (including signs of abdominal pain and other non IgE mediated allergies). Abdominal pain was only reported by one child in the BE group. Adjusting for total consumption of BE in our final analysis made no difference to the outcome.
- As was common practise when we designed this trial, to test for sustained unresponsiveness, participants in the trial had a 4-week break from BE prior to the end of study PE challenge. This may have affected the outcome of the trial compared with an OFC at the end of the 6 month period of BE ingestion.
- Ingestion of BE does not alter the resolution of raw egg allergy (i.e. the study finding of no effect is true). A population based study of 2 year-old BE tolerant, egg allergic children demonstrated frequent ingestion of BE was associated with earlier resolution of egg allergy [24]. Once BE intolerant children developed BE tolerance they were as likely to gain tolerance to regular egg as children initially tolerant to BE [24]. This may reflect a phenotype outgrowing their egg allergy more quickly than those self-limiting their BE intake.

There are no reports of controlled studies considering the effects of BE in the diets of older cohorts of egg allergic children, and such studies are warranted as BE may modulate the immune system in children with more resistant phenotypes of egg allergy.

## Conclusion

Our study suggests that short term, regular consumption of BE by BE tolerant 1 to 5 year old children with IgE mediated raw egg allergy may not induce, accelerate or slow development of tolerance to raw egg in this selected population. Trials with larger sample sizes are required to further test this hypothesis.

## Appendix 1: Methods

### Skin prick testing

To assess sensitisation to egg allergens, the children were skin prick tested to the common egg allergens, according to standard methods [35]. The allergen extracts used were egg white (Alyostal # 143, AUST R32582)(1:20 w/v), egg yolk (Alyostal # 144, AUST R32582)(1:20 w/v), (The Link Group/Stallergenes, Suburb, NSW), whole egg (ALK-Abello USA) (1:100 w/v), ovalbumin (ALK–Abello Spain; Allergen # 6.22)(1:20 w/v), and ovomucoid (ALK-Abello Spain; Allergen # 6.23)(1:20 w/v), (Australasian Medical and Scientific Ltd, Suburb, NSW). The negative control used was 50% (w/v) glycerin/saline (Holister-Steir Laboratories, Spokane, WA, USA) and the positive control was histamine phosphate (10 mg/ml, B 0911308) supplied by The Department of Pharmacy, Royal Adelaide Hospital, Adelaide, South Australia. A positive skin prick test to an allergen was defined as a mean of two perpendicular wheal diameters of 3 mm or greater in size than the mean wheal of the negative control site at 15 min.

### Measurement of antigen specific ige

Serum whole egg, egg white, ovalbumin and ovomucoid specific IgE and whole egg specific IgG4 concentrations in plasma were measured using the Phadia CAP system by the Department of Immunopathology, SA Pathology at the Women’s and Children’s Hospital, using NATA accredited methodologies as per SA Pathology protocols.

## Appendix 2 Immunological methodology

Immune memory development was assessed by CD45RA/CD45RO, and staining with CCR7 allowed assessment of changes in effector and central memory to be detected. To further assess immune activation peripheral blood mononuclear cells (PBMCs) sampled at baseline and the end of the intervention were incubated with OVA or OVM and assessed for T cell CD69 expression and cytokine excretion from the PBMCs was also measured to assess changes in the Th1/Th2 balance.

Peripheral blood samples were collected at the screening and 7 month visits and processed immediately. Plasma was collected after centrifugation and stored at −80C for determination of serum specific IgE/IgG4 levels. Peripheral blood mononuclear cells (PBMCs) were isolated by Ficoll-Hypaque (Alexis-Shield, Oslo, Norway) gradient centrifugation and isolated cells cryopreserved in 80% heat inactivated FCS (Sigma-Aldrich, Sydney, Australia) and 20% dimethyl sulfoxide (Ajax Finechem, Taren Point, NSW, Australia). Cell number and viability was assessed by Trypan Blue.

For the cell culture experiments, PBMCs (10^6^ cells/ml) were cultured with 100 μg/ml egg allergens OVA and OVM (Sigma-Aldrich, Sydney, Australia) for 5 days at 37 °C and 5% CO2. Cells cultured with AIM-V medium (Invitrogen, Life Technologies Sydney, Australia) alone served as a no-antigen control, and phytohemagglutinin-L (PHA-L) (1 ug/ml) (Roche Diagnostics, Australia or Remel, KS, USA) was used as the positive control. At day 3 (for PHA-L stimulated cells) and day 5 (for OVA, OVM and no antigen control) all available cells were harvested and analysed. Cells were centrifuged at 300xg for 5 min and the resulting supernatants collected and stored at −80 °C for cytokine analysis. The cell pellet was then equally divided into FACS tubes for labelling with mouse anti human conjugated monoclonal antibodies specific for cell surface antigens. Combinations of fluorophores PE, PE-Cy7, FITC, APC, APC-Cy7 or PerCP were used to assess the phenotype of the cells. All antibodies (except CCR7) were purchased from BD Biosciences (San Jose, CA, USA). CCR7 was purchased from (Miltenyi Biotec, Auburn, SD, USA). Cells were acquired on a BD Biosciences FACS Canto flow cytometer (Becton Dickinson, CA, USA). Isotype controls were used to set up the instrument and the positive gating, and these settings were maintained throughout. After selecting a lymphocyte gate based on forward and side-scatter characteristics, events within the lymphocyte gate were analysed using BD FacsDiva™ software version 6.1.3 (BD Biosciences, San Jose, CA, USA).

For the immunophenotyping cells were stained at baseline for CD4, CD8, CD14, CD19 and HLA DR. To assess activation, cells were stained with CD69 at baseline and after incubation with OVA and OVM and to assess memory markers cells were stained with CD45RO, CCR7, CD27 and CD28.

The cytokine concentration in supernatants collected on days 3 and 5 after incubation with OVA or OVM was assessed using a BD Cytometric Bead Array Human Inflammatory Cytokine Kit (IL-8, Il-1, IL-6, TNF, IL-12 and IL-10) and individual cytokines IL-4, IL-5 and IFNγ were measured using the BD Biosciences enhanced sensitivity flex sets for Human IL-4, Human IL-5 and Human IFNγ and a BD Cytometric Bead Array (CBA) Human enhanced Sensitivity Master Buffer Kit (BD Biosciences, San Jose, CA, USA). (Minimum levels of detection: IL-8, 2.6 pg/ml; IL-1, 7.2 pg/ml; IL-6 2.5 pg/ml; IL-10 3.3 pg/ml; TNF, 3.7 pg/ml; IL-12, 1.9 pg/ml). Beads were acquired on a BD Biosciences FACS Canto flow cytometer (Becton Dickinson, San Jose, CA, USA) and analysed using BD FacsDiva^TM^ software version 6.1.3 (BD Biosciences, San Jose, CA, USA).

## Appendix 3: Results of screening baked egg oral food challenges

**Table 3:** Screening baked egg oral food challenge outcomes

Outcome of oral food challenge	N (%)
Pass	48 (57.8%)^a^
Fail	26 (31.3%)
Suboptimal test	9 (10.8%)^b^
Average quantity of baked egg consumed before ceasing the OFC (grams)^c^	
Passed OFCs	9.0 (1.4) g
Failed OFCs	4.4 (2.8) g
Suboptimal OFCs	1.6 (1.3) g
Stop criteria	
Number of children reaching >1 stop criterion	7 (26.9%)
I. Skin	12 (46.2%)
A: Erythematous Rash	0 (0.0%)
B: Pruritus	0 (0.0%)
C: Urticaria/Angioedema	10 (38.5%)
D: Appearance of new skin rash	2 (7.7%)
II. Upper Respiratory	8 (34.8%)
A: Sneezing/Itching	0 (0.0%)
B: Nasal Congestion	1 (3.8%)
C: Rhinorrhoea	0 (0.0%)
D: Laryngeal	3 (11.5%)
III. Lower Respiratory	0 (0.0%)
IV. Gastrointestinal	15 (57.7%)
A: Subjective complaints (>18 months old)	7 (26.9%)
B: Objective complaints	9 (34.6%)
V. Cardiovascular	0 (0.0%)
Treatment	
None	2 (7.7%)
Cetirizine	24 (92.3%)
Adrenaline	2 (7.7%)
Prednisolone	2 (7.7%)

Comments regarding Table 2: ^a^51.8% (43/83) were BE tolerant, egg allergic (8/48 BE tolerant children had raw egg OFC, 6/8 tolerated raw egg and were excluded from final analysis). ^b^1/9 with suboptimal BE OFC tolerated a raw egg OFC. ‘Stop criterion’ results do not add to 100% as 7/27 children failing the BE OFC met > one criterion. ^c^1/8 of a muffin contained 1.25 g of baked egg (0.2 g egg protein), mean (SD)

## Abbreviations

BE: Baked egg
EW: Egg white
EY: Egg yolk
Ig: Immunoglobulin
OFC: Oral food challenge
OVA: Ovalbumin
OVM: Ovomucoid
SOTI: Specific oral tolerance induction
SPT: Skin prick test
WE: Whole egg
